# Auditory connections and functions of prefrontal cortex

**DOI:** 10.3389/fnins.2014.00199

**Published:** 2014-07-23

**Authors:** Bethany Plakke, Lizabeth M. Romanski

**Affiliations:** Department of Neurobiology and Anatomy, University of Rochester School of Medicine and DentistryRochester, NY, USA

**Keywords:** monkey, working memory, acoustic, frontal lobe

## Abstract

The functional auditory system extends from the ears to the frontal lobes with successively more complex functions occurring as one ascends the hierarchy of the nervous system. Several areas of the frontal lobe receive afferents from both early and late auditory processing regions within the temporal lobe. Afferents from the early part of the cortical auditory system, the auditory belt cortex, which are presumed to carry information regarding auditory features of sounds, project to only a few prefrontal regions and are most dense in the ventrolateral prefrontal cortex (VLPFC). In contrast, projections from the parabelt and the rostral superior temporal gyrus (STG) most likely convey more complex information and target a larger, widespread region of the prefrontal cortex. Neuronal responses reflect these anatomical projections as some prefrontal neurons exhibit responses to features in acoustic stimuli, while other neurons display task-related responses. For example, recording studies in non-human primates indicate that VLPFC is responsive to complex sounds including vocalizations and that VLPFC neurons in area 12/47 respond to sounds with similar acoustic morphology. In contrast, neuronal responses during auditory working memory involve a wider region of the prefrontal cortex. In humans, the frontal lobe is involved in auditory detection, discrimination, and working memory. Past research suggests that dorsal and ventral subregions of the prefrontal cortex process different types of information with dorsal cortex processing spatial/visual information and ventral cortex processing non-spatial/auditory information. While this is apparent in the non-human primate and in some neuroimaging studies, most research in humans indicates that specific task conditions, stimuli or previous experience may bias the recruitment of specific prefrontal regions, suggesting a more flexible role for the frontal lobe during auditory cognition.

## Introduction

Connections from the auditory cortex to the frontal lobes mediate a number of functions including language, object recognition and spatial localization. Discerning what types of auditory information reaches the frontal cortex, where that auditory input originates, and how information is utilized by the frontal lobes for complex behaviors, such as communication, is a fundamental question of neuroscience.

**Figure 1:**
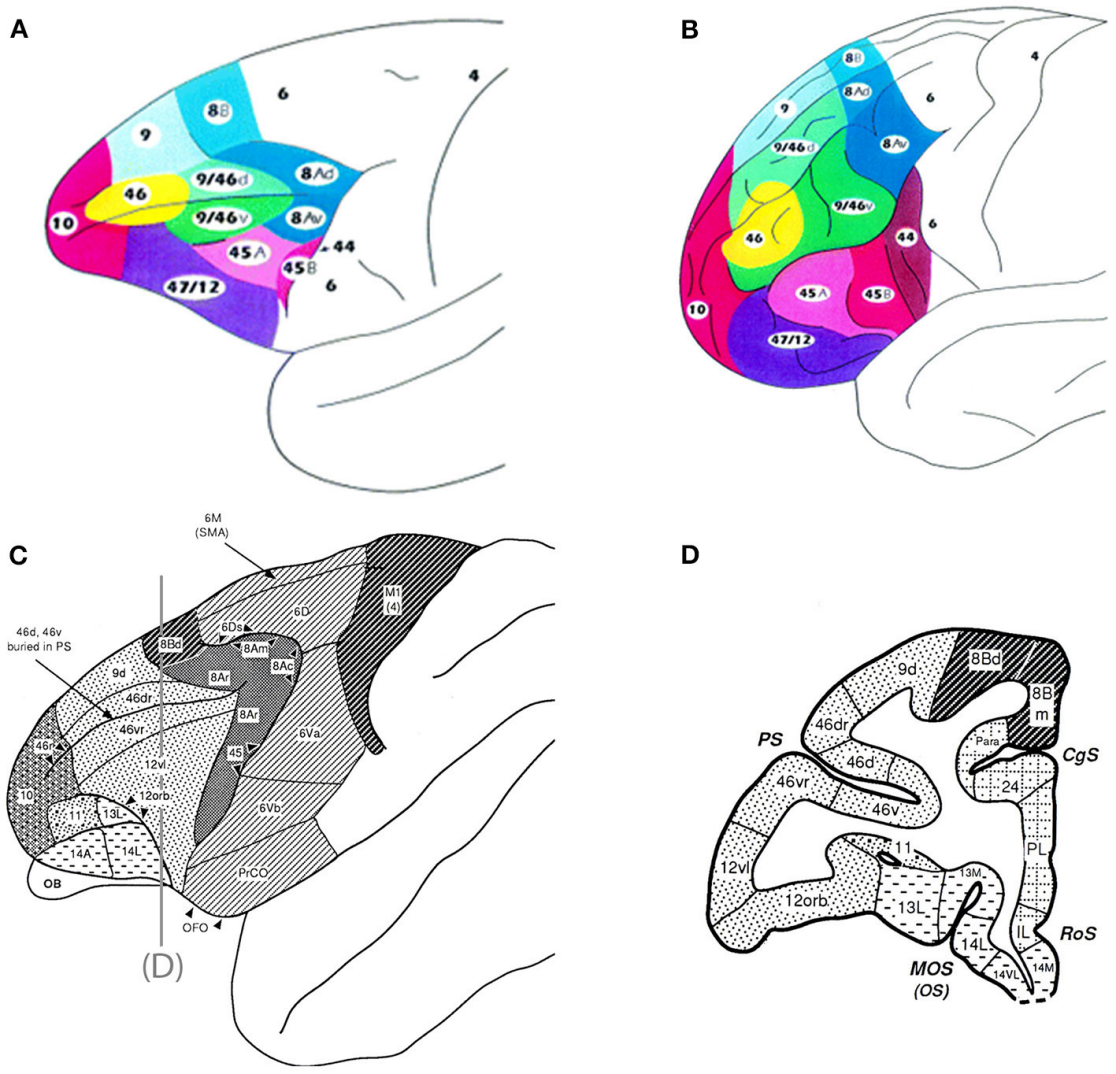
**Top panel are schematics of the lateral and frontal surfaces of the monkey (A) and human (B) brain from Petrides and Pandya (2002)**. Bottom panel are schematics illustrating area 12 vl (now referred to as 12/47) and 12 orb **(C)** and a coronal section **(D)** depicting these regions from Preuss and Goldman-Rakic (1991). Inset diagram is the lower part of arcuate sulcus to show cytoarchitectonic areas within the banks of the sulcus. Used/modified with permission from Petrides and Pandya (2002) and Preuss and Goldman-Rakic (1991).

The frontal cortex is a heterogeneous region with multiple functional subdivisions, including the prefrontal cortex, which lies in the anterior frontal lobe and consists of medial, lateral, and orbital subdivisions. This review will focus on the lateral prefrontal cortex including the dorsolateral regions (DLPFC) (areas 8, 46, and 9) and the ventrolateral regions (VLPFC) (areas 12/47, 45, and 12 orbital) (Figure 1). Possible auditory functions and connections of frontal pole, medial and orbital areas of the frontal lobe are described elsewhere including Medalla and Barbas (2014).

The frontal lobe is well-known for its role in speech and language processes and executive functions that include working memory, planning, and decision making (Fuster, 2008). Early lesion studies indicated that lesions of prefrontal cortex caused impairments in delay response, delay spatial alternation, and delay object alternation tasks (Pribram et al., 1952; Mishkin and Pribram, 1955, 1956; Pribram and Mishkin, 1956; Mishkin et al., 1969). Later, more precise lesion studies implicated DLPFC in spatial and delay processes (Malmo, 1942; Mishkin, 1957; Passingham, 1975; Mishkin and Manning, 1978). In contrast, lesions of VLPFC resulted in impaired performance in non-spatial tasks and implicated VLPFC in object recognition (Mishkin and Manning, 1978). In the last two decades there have been a wealth of neuroimaging studies in human subject and single-unit recording studies in non-human primates, which confirm a role in working memory for the prefrontal cortex (Funahashi et al., 1993; Awh et al., 1996; McCarthy et al., 1996; Miller et al., 1996; Owen et al., 1996; Courtney et al., 1997; D'Esposito et al., 2000; Fuster et al., 2000; Bunge et al., 2003; Postle et al., 2003; Bor et al., 2004; Rowe et al., 2008). Unfortunately most neurophysiology studies utilize visual working memory paradigms. Therefore, while these studies have shed light on the neuronal mechanisms underlying prefrontal visual information processing and visual memory, there is much less known about prefrontal processing of auditory information. Fortunately, the past decade has seen several advances in our understanding of the organization of the primate auditory cortical system and how this system, is critical for speech, auditory attention, and multisensory integration. These advances have made it possible and necessary to investigate the pathways that bring auditory information to the prefrontal cortex and the neural mechanisms which underlie auditory cognition.

### Auditory connections of the frontal lobes

Historically, anatomical tract tracing and lesion degeneration studies provided evidence that presumptive auditory cortical regions send projections to prefrontal cortex. One general principal observed in these studies of prefrontal-auditory connections is the rostro-caudal topography (Pandya and Kuypers, 1969; Chavis and Pandya, 1976; Petrides and Pandya, 1988; Seltzer and Pandya, 1989; Barbas, 1992; Romanski et al., 1999a,b). Reciprocal connections are apparent between the caudal STG and caudal PFC, including caudal (dorsal) area 46, periarcuate area 8a and the inferior convexity, or ventral prefrontal cortex—areas 12 and 45 (Petrides and Pandya, 1988; Barbas, 1992). In addition, middle and rostral STG are reciprocally connected with rostral 46 and area 10 and orbito-frontal areas 11 and 12 (Pandya and Kuypers, 1969; Pandya et al., 1969; Chavis and Pandya, 1976). Furthermore, studies noted projections from the anterior temporal lobe to orbital and medial prefrontal cortex and the frontal pole (Petrides and Pandya, 1988; Barbas, 1993; Carmichael and Price, 1995; Hackett et al., 1999; Romanski et al., 1999a).

While these studies inform us of the existence of temporal prefrontal connectivity they do not indicate which of these connections carries acoustic information. To understand the flow of auditory information to the prefrontal cortex, it is necessary to know what parts of the temporal lobe are, in fact auditory responsive. Progress in defining the connections and areal organization of the auditory cortex was greatly accelerated by advancements in auditory cortical neurophysiology and neuroanatomy. First, Rauschecker and colleagues delineated the physiological boundaries of auditory cortical core and belt regions (Rauschecker et al., 1995, 1997). These studies provided the first electrophysiological evidence for three separate tonotopic regions in the non-primary lateral belt cortex (AL, ML, and CL) (antero-lateral belt, middle-lateral belt, caudal-lateral belt cortex respectively) with frequency reversals separating them. Compared with primary auditory cortical neurons, which readily respond to relatively simple acoustic elements, such as pure tones, neurons of the lateral belt association cortex prefer complex stimuli including band-passed noise and vocalizations (Rauschecker et al., 1995, 1997). Simultaneous advances in anatomical organization confirmed and extended these findings. Several groups showed that primary and non-primary auditory cortex could be distinguished on the basis of differential staining for the calcium binding protein parvalbumin along with cytoarchitectonic changes (Morel et al., 1993; Jones et al., 1995; Kosaki et al., 1997; Hackett et al., 1998). These combined physiological and anatomical studies made it possible to recognize individual boundaries of the auditory cortical system and showed its organization to consist of a primary, or core, region composed of potentially two areas, AI and R, surrounded by and connected to, a medial and lateral belt of secondary auditory association cortex with a lower density of parvalbumin staining (Morel et al., 1993; Jones et al., 1995; Kosaki et al., 1997; Hackett et al., 1998). A third zone lying adjacent to the lateral belt is the parabelt auditory cortex. Further distinctions between the core and belt, and the belt and parabelt have been based on myeloarchitectonic, and connectional differences. Recent neurophysiological studies have examined the more complex auditory and multisensory responses of the belt (Ghazanfar et al., 2005; Kuśmierek et al., 2012), the rostral superior temporal gyrus (STG) (Kikuchi et al., 2010; Tsunada et al., 2012; Scott et al., 2013, SFN; Perrodin et al., 2014), and the cortex of the superior temporal sulcus (STS) (Ghazanfar et al., 2008; Kikuchi et al., 2010).

**Figure 2:**
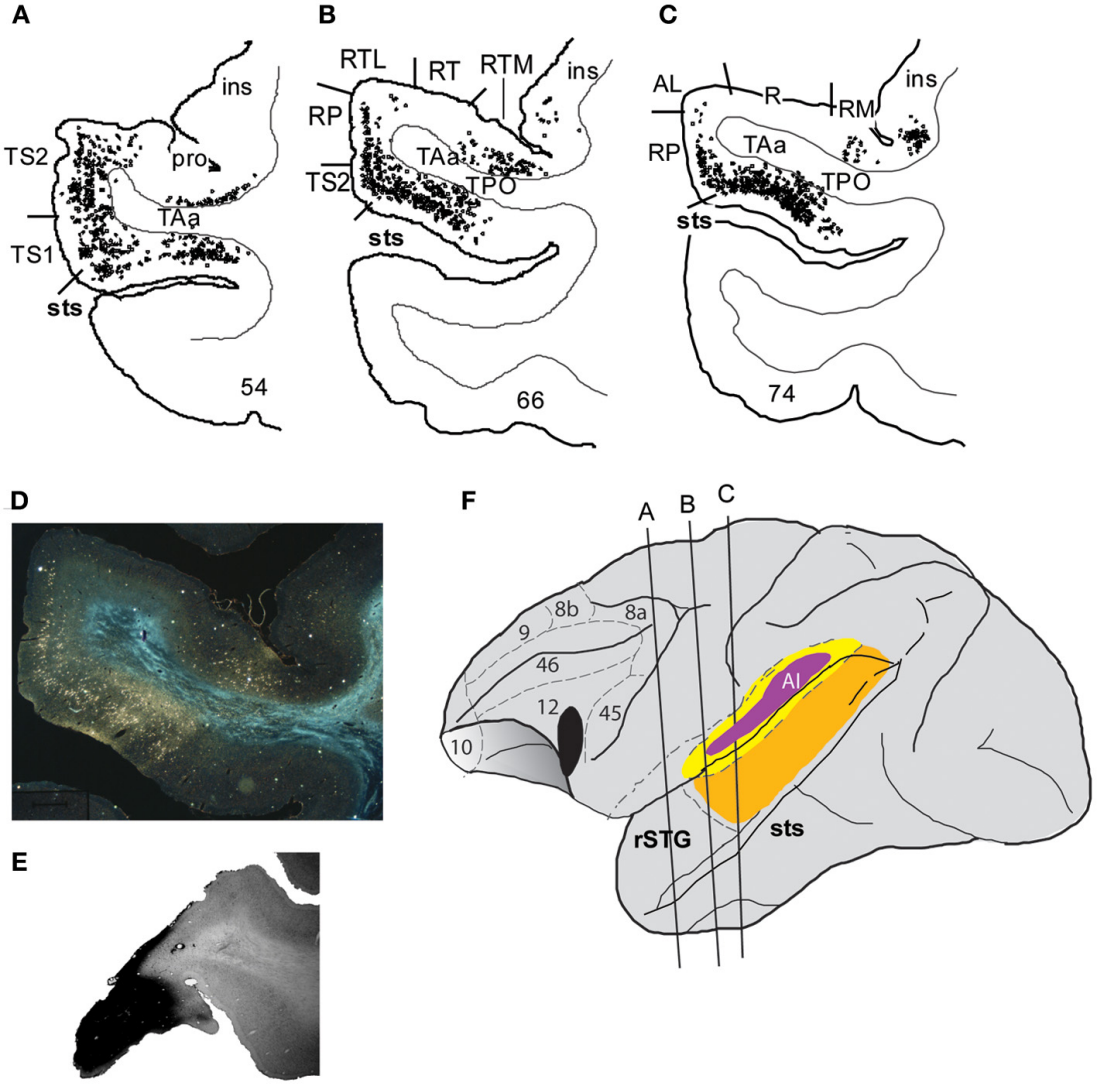
**Connections of VLPFC with auditory cortex**. An injection of WGA-HRP shown previously (Romanski et al., 1999a) illustrates the density of connections of VLPFC with auditory cortical regions in the belt, parabelt and superior temporal sulcus as well as the rostral temporal lobe. Coronal sections through the temporal lobe are shown in **(A–C)** with black dots illustrating the location of retrogradely labeled cells. Labeling is heaviest in the superior temporal sulcus regions TPO and TAa, moderate in the parabelt and lighter in the lateral belt. **(D)** A photomicrograph from a temporal lobe section adjacent to that shown in **(B)**. **(E)** Is a photomicrograph of the prefrontal cortex section containing the injection site for this injection which was located in the ventral part of area 12/47. **(F)** Portrays the location of the injection site in VLPFC and the locations of the coronal sections from A–C on a lateral schematic of the macaque brain. Adapted from Romanski et al. (1999a).

**Figure 3:**
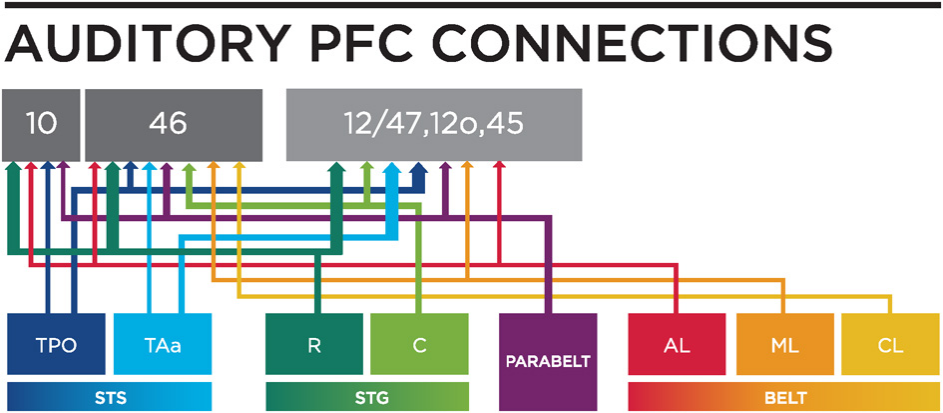
**A circuit diagram summary of auditory inputs from temporal lobe areas to the PFC, from anatomical studies (including: Petrides and Pandya, 1988; Seltzer and Pandya, 1989; Hackett et al., 1999; Romanski et al., 1999a,b) discussed in this review**. Thicker lines represents stronger connections. R, Rostral; C, Caudal; STS, Superior Temporal Sulcus; STG, Superior Temporal Gyrus; AL, Antero-lateral; ML, Middle-lateral; CL, Caudal-lateral; TPO, temporal parieto-occiptal area; TAa, Temporal area.

Two relevant studies followed on the heels of this revised characterization of auditory core and belt regions and described prefrontal-auditory connections in the context of these defined core, belt and parabelt regions (Hackett et al., 1999; Romanski et al., 1999a). A series of >15 tracer injections into discrete cytoarchitectonic regions of the prefrontal cortex showed that the rostral, orbital and ventrolateral areas of the prefrontal cortex are reciprocally connected with the rostral STG, the rostral belt (areas AL and anterior ML) and the rostral parabelt, whereas caudal principalis and some dorsolateral regions (46, 8, 9) of the prefrontal cortex are reciprocally connected with the caudal belt (caudal ML and CL) and caudal parabelt (Romanski et al., 1999a). Importantly, projections to the PFC from higher order cortical auditory regions such as parabelt and STS were more robust than the early auditory cortical regions such as the lateral belt, suggesting a cascade of lighter to stronger projections to the prefrontal cortex from early to late auditory processing regions (Figure 2), (Hackett et al., 1999; Romanski et al., 1999a). Furthermore, the ventrolateral prefrontal cortex (VLPFC) was shown to have a very dense reciprocal connection with the dorsal bank of the STS including areas TPO (temporal parieto-occipital junction), and TAa (temporal area a) (Romanski et al., 1999a; Figures 2, 3).

While these anatomical studies suggest that the PFC receives auditory information, since afferents from the auditory belt and parabelt terminate in PFC, more direct evidence that projections are carrying acoustic information is obtained when anatomical and physiological methods are combined. In one such study, Romanski et al. (1999b) recorded auditory responses from lateral belt auditory areas AL, ML, and CL and placed injections of anatomical tracers into these physiologically defined regions. These connections were topographically organized such that projections from AL typically involved the frontal pole (area 10), the rostral principal sulcus (area 46), the inferior convexity (areas 12/47 and 45) and the lateral orbital cortex (areas 11, 12o). In contrast, projections from area CL targeted the dorsal periarcuate cortex (area 8a, frontal eye fields) and the caudal principal sulcus (area 46), and a small connection with caudal inferior convexity (areas 12/47 and 45) and, in two cases, premotor cortex (area 6d). These highly specific rostrocaudal topographical frontal-temporal connections suggest the existence of separate streams of auditory information that targeted previously identified visual domains in the prefrontal cortex. One pathway, originating in CL, targets caudal DLPFC; the other pathway, originating in AL, targets rostral prefrontal cortex and VLPFC. Previous studies have designated these regions in the frontal lobe as being involved in visuo-spatial (DLPFC) and visual object (VLPFC) processing based on physiological responses to visual stimuli (Wilson et al., 1993; O'Scalaidhe et al., 1997). Thus, it is possible the pathways originating from anterior and posterior auditory belt and parabelt cortices are analogous to the “what” and “where” streams of the visual system and that auditory functions in VLPFC and DLPFC could also be object and spatially based, respectively.

Further exploration of prefrontal auditory connections has focused on the VLPFC following the discovery of auditory responsive neurons in VLPFC, (Romanski and Goldman-Rakic, 2002). Anatomical connections of VLPFC regions with auditory belt, parabelt and rostral STG have been confirmed in other anatomical studies (Gerbella et al., 2010; Saleem et al., 2014) though clarification on whether area 45 or 12/47 receives greater auditory inputs is still needed (Romanski, 2012). Previous examination of responses in area 45 and the gradation of visual responses from the frontal eye fields located just dorsal to it argue in favor of stronger visual inputs to area 45 (Webster et al., 1994; Bullier et al., 1996; O'Scalaidhe et al., 1997). Previous cytoarchitectonic studies of VLPFC in *M. Mulatta* differ with the recent studies cited by Gerbella et al. (2010) and Saleem et al. (2014). Our organization of VLPFC is based on parcellations mainly by Preuss and Goldman-Rakic (1991) with additional studies by Carmichael and Price (1995), Medalla and Barbas (2014), Price (2008), Barbas (1988), and Saleem et al. (2008). Furthermore, we maintain that characterization of VLPFC must be accomplished with both anatomical and physiological data as stated above. Cytoarchitectonic boundaries vary across the different the studies we have referenced. Preuss and Goldman-Rakic (1991) show a much smaller boundary for area 45 while Saleem et al. (2014) shows it to be much larger. Gerbella et al. (2010) and Petrides and Pandya (2002) show differences in their parcellation of area 12. These differences confirm that additional studies combining neurophysiology and anatomical methods are needed to understand the organization of the frontal lobe in general, and VLPFC specifically.

**Figure 4:**
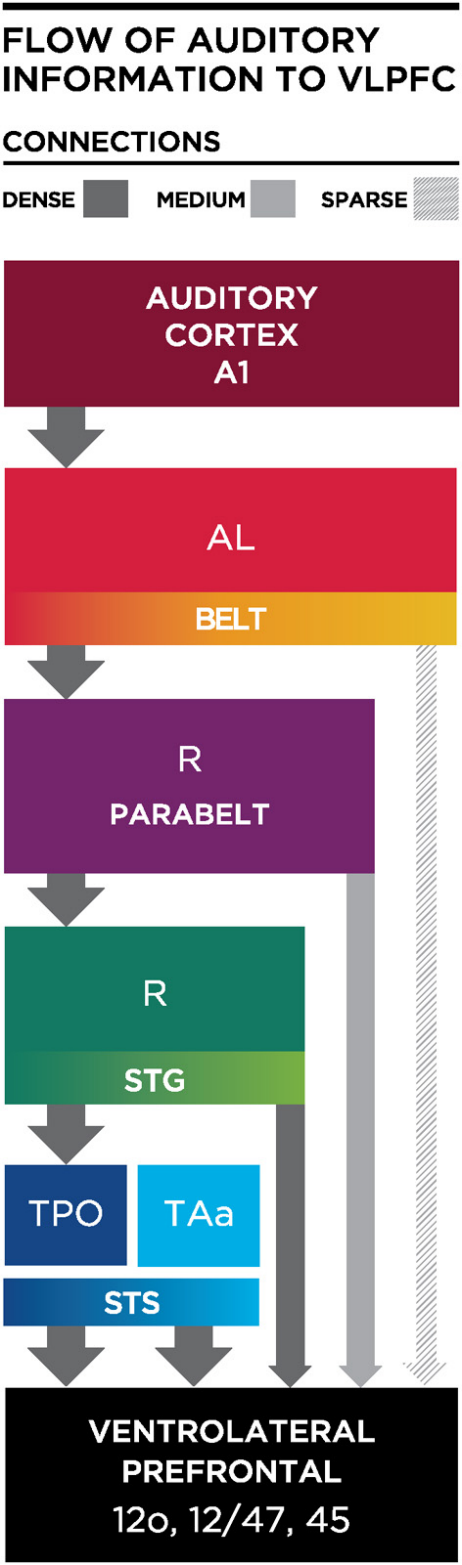
**Schematic diagram illustrating the flow of information from the auditory cortex to the VLPFC**. Thick and dark gray arrows illustrate dense projections from STS, with less dense projections arriving from parabelt and lateral belt regions. AL, Antero-lateral; R, Rostral; STS, Superior Temporal Sulcus; STG, Superior Temporal Gyrus; TPO, temporal parieto-occiptal area; TAa, Temporal area.

One principle that has emerged from anatomical studies is that a cascade of afferents reaches the VLPFC (Figure 4). The densest projections to VLPFC originate from the STS and as-yet-uncharacterized regions of the rostral STG, while the parabelt provides a moderate innervation of rostral and ventrolateral regions (area 12/47 and area 12o). In contrast, the anterior and middle auditory belt cortex provides only a modest input to VLPFC (Hackett et al., 1999; Romanski et al., 1999a,b; Figures 3, 4), though their input may arrive earliest due to fewer synaptic junctions. This is in agreement with the notion that our association cortical regions receive highly processed information about a sensory stimulus after it has undergone transformations through earlier sensory cortical regions.

### Physiological responses of neurons in PFC

Prior to 2000, responses to acoustic stimuli of a non-spatial nature were sporadically noted across a widespread region of the frontal lobe in Old and New World primates (Newman and Lindsley, 1976; Benevento et al., 1977; Wollberg and Sela, 1980; Tanila et al., 1992, 1993; Watanabe, 1992; Bodner et al., 1996). Several of these studies used auditory stimuli in combination with visual stimuli as task elements but did not systematically explore the selectivity of auditory responsive cells (Ito, 1982; Vaadia et al., 1986, 1989; Watanabe, 1992). Despite reports of responses to complex stimuli including clicks, environmental sounds and vocalizations, the prior neurophysiological recordings in the frontal lobe of non-human primates failed to demonstrate a discrete clustering of auditory cells indicative of an auditory responsive domain (Newman and Lindsley, 1976; Tanila et al., 1992, 1993). Building on the connectional studies which predicted an auditory-responsive region in VLPFC (Romanski et al., 1999a,b), neurophysiological studies investigated the responses of lateral PFC neurons. Romanski and Goldman-Rakic (2002), described a discrete auditory responsive region in the macaque prefrontal cortex in which a region of VLPFC had neurons which responded to a variety of complex acoustic stimuli including species-specific vocalizations. The auditory responsive region was small (4 × 4 mm) and was localized to the VLPFC, mostly area 12/47 and potentially area 45 (Romanski and Goldman-Rakic, 2002). Further analysis showed that prefrontal neurons typically responded to stimuli that were acoustically similar (Romanski et al., 2005). Specifically neurons responded to species-specific vocalizations that had a similar acoustic morphology and not a similar behavioral referent, (Romanski et al., 2005). Analysis of the classification of the vocalizations with a hidden Markov model (HMM), showed that the HMM was more effective at discriminating among the call classes than previous methods, reaching a classification performance of almost 75% correct. Furthermore the complex responses of prefrontal neurons to these sounds could be predicted as linear functions of the probabilistic output of the HMM (Averbeck and Romanski, 2006).

The auditory responsive region in VLPFC lies adjacent to a region where visually responsive neurons, face cells and face-responsive patches have been localized (O'Scalaidhe et al., 1997, 1999; Tsao et al., 2008). Thus, the idea that VLPFC neurons might be responsive to both vocalization and faces is hardly surprising. VLPFC, as mentioned previously, receives afferents from both auditory and visual portions of the temporal lobe as well as a robust innervation from the multisensory area TPO in the dorsal bank of the STS (Barbas, 1988; Romanski et al., 1999a,b). A study by Benevento et al. (1977) found neurons in VLPFC (area 12o) that were responsive to simple auditory and visual stimuli (clicks and light flashes), and, as demonstrated with intracellular recordings, at least some of these interactions were due to convergence on single cortical cells. Using species-specific vocalizations and their accompanying facial gestures, Romanski and colleagues demonstrated multisensory responses to simultaneously presented faces and vocalizations in VLPFC neurons (Sugihara et al., 2006). Sugihara et al. (2006) further characterized multisensory responses as enhanced or suppressed. Multisensory neurons accounted for about half the recorded population with ~4% unimodal auditory responses and ~50% unimodal visual responses, suggesting that a large proportion of VLPFC neurons are likely to be multisensory if tested properly. Since the region of VLPFC where multisensory neurons are located overlaps extensively with the location of previously characterized auditory responses, it is probable that previous studies which examined either unimodal auditory or unimodal visual functions included multisensory cells in their populations.

The anatomical studies described above have shown that the auditory responsive regions in VLPFC receives very dense innervation from areas TPO and TAa multisensory zones on the dorsal bank of the STS (Romanski et al., 1999a; Figures 2, 4), with moderate projections from the rostral STG and parabelt and lighter inputs from the anterior and middle belt (AL and ML) to VLPFC. Thus, VLPFC neurons may receive acoustic afferents from early (belt) or late (TPO/rostral STG) regions of the auditory cortical hierarchy. It is possible that the specific pattern of afferent input may dictate the types of neurophysiological responses found in VLPFC. The fact that neurons in VLPFC exhibit a wide range of response latencies to auditory stimuli (30–330 ms) also supports this concept of heterogeneous afferents (Romanski and Hwang, 2012). For example, a small number of auditory responsive neurons have extremely fast latency responses, these cells could be receiving inputs from early auditory cortical areas (Romanski et al., 2005; Romanski and Hwang, 2012) with narrow selectivity and phasic onsets to acoustic stimuli. It is possible that these feature-sensitive, rapid onset responses could arise from early auditory cortex such as the anterior belt region AL which is known to project sparsely to this region and would arrive first. In contrast, neurons which respond to combinations of complex acoustic features, or more generally to task variables may be more likely to receive afferents from parabelt and rostral STG which would be several synapses away from VLPFC and would presumably take longer and provide more highly processed information about an auditory object. Finally, multisensory responses in VLPFC could arise as a *de novo* integration of inputs from auditory belt, parabelt or rostral STG and extrastriate visual cortical areas such as TE. Alternatively multisensory VLPFC responses could originate from multisensory cells of TPO or TAa on the dorsal bank of the STS, which send dense projections to VLPFC. Multisensory responses in VLPFC have longer latencies than unimodal auditory response latencies measured in the same cells (multisensory response range 50–490 ms; Romanski and Hwang, 2012).

### Localization of auditory function in DLPFC and VLPFC: animal studies

As reviewed above the frontal cortex receives afferents from early and late auditory cortical processing stations allowing frontal lobe neurons to detect and discriminate auditory stimuli (Ito, 1982; Watanabe, 1992; Romanski and Goldman-Rakic, 2002; Poremba et al., 2004), or to be remembered during auditory working memory processes (Plakke et al., 2013a). Divergent processing pathways conforming to ventral and dorsal “what” and “where” streams, respectively, originate in the belt and parabelt auditory cortex and terminate in VLPFC and DLPFC regions as described above. DLPFC receives information from caudal auditory regions, which have been shown to preferentially process auditory location information and VLPFC receives input from rostral auditory regions that show a greater preference for type of stimuli (Romanski et al., 1999b; Rauschecker and Tian, 2000; Tian et al., 2001; Kuśmierek et al., 2012). Based on these anatomical connections it has been proposed that DLPFC is primarily involved in spatial processing while VLPFC may be preferentially involved in object processing.

This traditional division of labor between dorsal and ventral prefrontal regions is supported by some neurophysiology studies. Early studies demonstrated that DLPFC neurons were preferentially responsive when acoustic stimuli were presented from specific directions (Azuma and Suzuki, 1984) or when animal subjects localized auditory or visual stimuli (Vaadia et al., 1989). In latter studies which focused on working memory processes, neurons in DLPFC were active during the mnemonic processing of auditory and visual location (Kikuchi-Yorioka and Sawaguchi, 2000; Artchakov et al., 2007). In both studies, a portion of DLPFC neurons were spatially selective during the delay for both auditory and visual cues.

**Figure 5:**
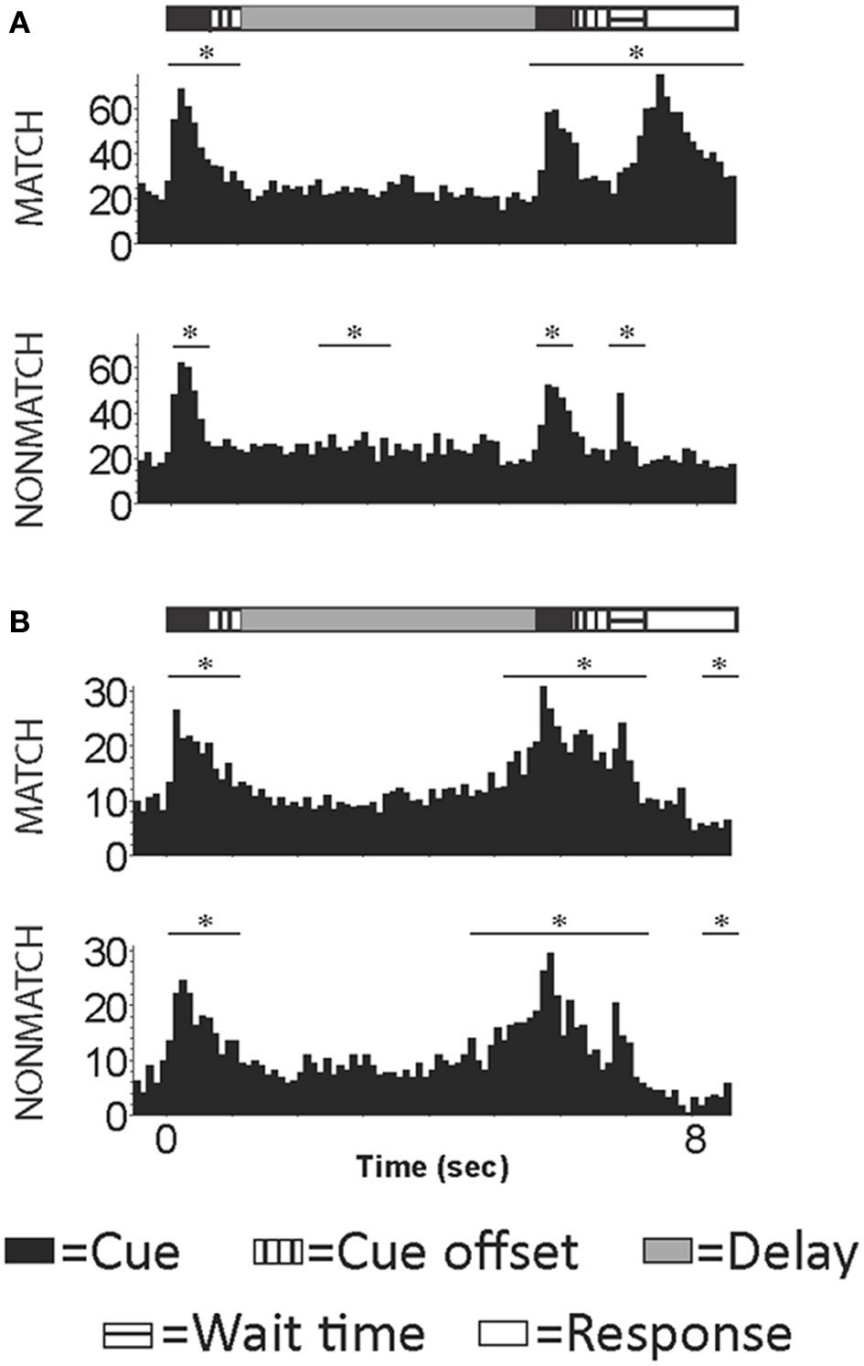
**Example cells with activity occurring during the presentation of the auditory sample, match/nonmatch and during the decision period of an auditory delayed match-to-sample task. (A)** An example cell with increased activity during the auditory cues, wait time and response periods for correct trials. **(B)** An example cell with increased firing rated during auditory cue and wait time periods for correct trials. Y-axis label is frequency (imp/s); bin = 100 ms; asterisk signifies significant change in firing rate from baseline.

However, other neurophysiological studies demonstrated that DLPFC neurons were active during non-spatial tasks. Studies by Watanabe (1992) showed that prefrontal neurons responded when tones were predictive of juice reward and Bodner et al. (1996) described auditory working memory cells in DLPFC during a task where a tone was paired with a color to predict reward. More recently, recordings during a non-spatial auditory delayed match-to-sample task demonstrated task related activity in neurons in both dorsal and ventral PFC (Plakke et al., 2013a). Prefrontal neurons responded to sound cues during both the sample and match/nonmatch presentations, and also during the delay, response, and reward periods of the task (Plakke et al., 2013a), (Figure 5). During this task, cells in this region appeared to be responsive to tracking when a relative stimulus is needed to be remembered or responded too. The general task responses of these neurons suggests that the role of the DLPFC in auditory working memory may be for rule representation or response control, as previously suggested in studies of visual working memory (Fuster et al., 1982; Miller et al., 1996; Iba and Sawaguchi, 2002; Warden and Miller, 2007). Together these studies suggest that the role of DLPFC in auditory memory may relate more to task and cognitive requirements than to acoustic stimulus encoding.

In contrast to the task related processes in DLPFC, neurophysiology in non-human primates suggest that VLPFC may perform both stimulus and task related processes. As described above, VLPFC contains neurons that are responsive to complex sounds including, species-specific vocalizations and human vocalizations (Romanski and Goldman-Rakic, 2002; Romanski et al., 2005), suggesting a role for VLPFC in auditory object processing. VLPFC involvement in auditory feature processing is supported by studies showing single-units that encode categories of vocalization call types (Averbeck and Romanski, 2004, 2006; Plakke et al., 2013b). Moreover, evidence that VLPFC cells are multisensory and respond to the simultaneous presentation of faces and their corresponding vocalizations strongly suggests a role in recognition and identity processing, a ventral stream function (Sugihara et al., 2006).

Several studies from Cohen and colleagues have examined neuronal responses in VLPFC during non-spatial auditory performance tasks (Cohen et al., 2006, 2007; Russ et al., 2008a; Tsunada et al., 2011). For example, VLPFC neurons were modulated during non-spatial auditory discrimination but showed no modulation during spatial auditory discrimination (Cohen et al., 2009). Further recordings over a large region of PFC which Cohen termed “vPFC” during categorization and decision making paradigms, demonstrate that prefrontal neuronal activity is correlated with behavioral choices (Russ et al., 2008b; Lee et al., 2009), although the location of these prefrontal neurons does not appear to overlap entirely with the ventrolateral PFC regions previously shown to be auditory responsive (Romanski et al., 2005). Nonetheless, inactivation studies are needed to determine whether VLPFC is essential in the performance of working memory or decision making tasks. Toward this end, a recent study by Plakke et al., (2013c, SFN) shows that transient inactivation of VLPFC impairs performance in an audiovisual working memory task and suggests an essential role in mnemonic processing when acoustic stimuli are involved. Thus, processing of auditory information in DLPFC may relate more to the task demands, while processing of auditory information in VLPFC is clearly related to auditory features and task demands.

### Processing of auditory information in the human dorsal and ventral PFC

The anatomical and neurophysiological studies performed in nonhuman primates delineate somewhat separable roles for dorsal and ventral frontal lobe regions. How these functional streams in nonhuman primates map onto auditory function in the human brain is, as yet, not completely clear. Although it is well known that speech and language functions rely on the cortex within the inferior frontal gyrus (IFG) neuroimaging studies have provided evidence that the human frontal lobe is also active during auditory discrimination (Zatorre et al., 1994), auditory detection (Benedict et al., 1998, 2002), auditory attention/oddball tasks (Stevens et al., 2000), auditory judgments (Zatorre et al., 1998), and auditory working memory (Anurova et al., 2003, 2001; Grady et al., 2008; Protzner and McIntosh, 2009). These studies have described discrete activations in DLPFC and VLPFC that are related to the type of information processed. For example, several imaging studies have described activations in DLPFC (superior frontal gyrus, superior frontal sulcus) during auditory spatial localization (Griffiths et al., 1998; Martinkauppi et al., 2000; Weeks et al., 2000; Lipschutz et al., 2002; Lutzenberger et al., 2002; Zatorre et al., 2002; Gaab et al., 2003; Leiberg et al., 2006). Conversely, VLPFC activation (IFG; BA 45,47), has been noted during auditory non-spatial processes, such as listening to melodies, attending pitch/rhythm, determining sound length, word/voice discrimination and auditory working memory (Zatorre et al., 1994, 1998; Platel et al., 1997; Linden et al., 1999; Pedersen et al., 2000; Alain et al., 2001; Kiehl et al., 2001; Muller et al., 2001; Kaiser et al., 2003; Maddock et al., 2003; Arnott et al., 2004; Rämä et al., 2004; Rämä and Courtney, 2005; Kaiser et al., 2009; Koelsch et al., 2009).

In addition, activation of DLPFC (Brodman's area 46/9) occurs during various complex working memory paradigms. For instance there were increases in activity in DLPFC when participants listened to numbers and made self-ordered choices (Petrides et al., 1993). Dorsolateral activity is also increased during studies of divided auditory attention (Benedict et al., 1998) as well as encoding of nonverbal sounds (Opitz et al., 2000). Taken together these studies suggest DLPFC may be recruited more frequently based on cognitive demands including the type of process that is necessary such as monitoring information in memory, encoding auditory information, as well as manipulation of spatial information.

In contrast, the IFG and related VLPFC regions are activated during phonological processing (Klein et al., 1995; Buchanan et al., 2000; Strand et al., 2008), semantic processing (Caplan et al., 2000; Burton et al., 2003), syntactic operations (Waters et al., 2003), naming objects (Tranel et al., 2003), word discrimination (Buchanan et al., 2000; Vaden et al., 2013), and directed auditory attention (Hill and Miller, 2010) reinforcing the connection of this region with language and auditory feature processing. Interestingly, there has also been activation within the IFG during nonverbal auditory stimulus detection (Linden et al., 1999; Kiehl et al., 2001; Maeder et al., 2001), nonverbal auditory discrimination (Zatorre et al., 1994; Muller et al., 2001), and auditory working memory (Kaiser et al., 2003). The activation of the more anterior regions of the IFG (areas 47 and 45) during nonverbal auditory sound detection, discrimination and auditory feature detection (Zatorre et al., 2004; Fecteau et al., 2005) suggests these areas may play a more fundamental role in auditory processing, paralleling the auditory responsive region that has been described in non-human primates (Romanski and Goldman-Rakic, 2002; Romanski et al., 2005). The role of VLPFC in general sound discrimination is also supported by its activation when listening to rhymes (Burton et al., 2003) and by the case of a patient with an inferior frontal lesion that was impaired on detecting modulated sounds (Griffiths et al., 2000).

### Verbal vs. non-verbal stimuli and cognitive requirements

**Figure 6:**
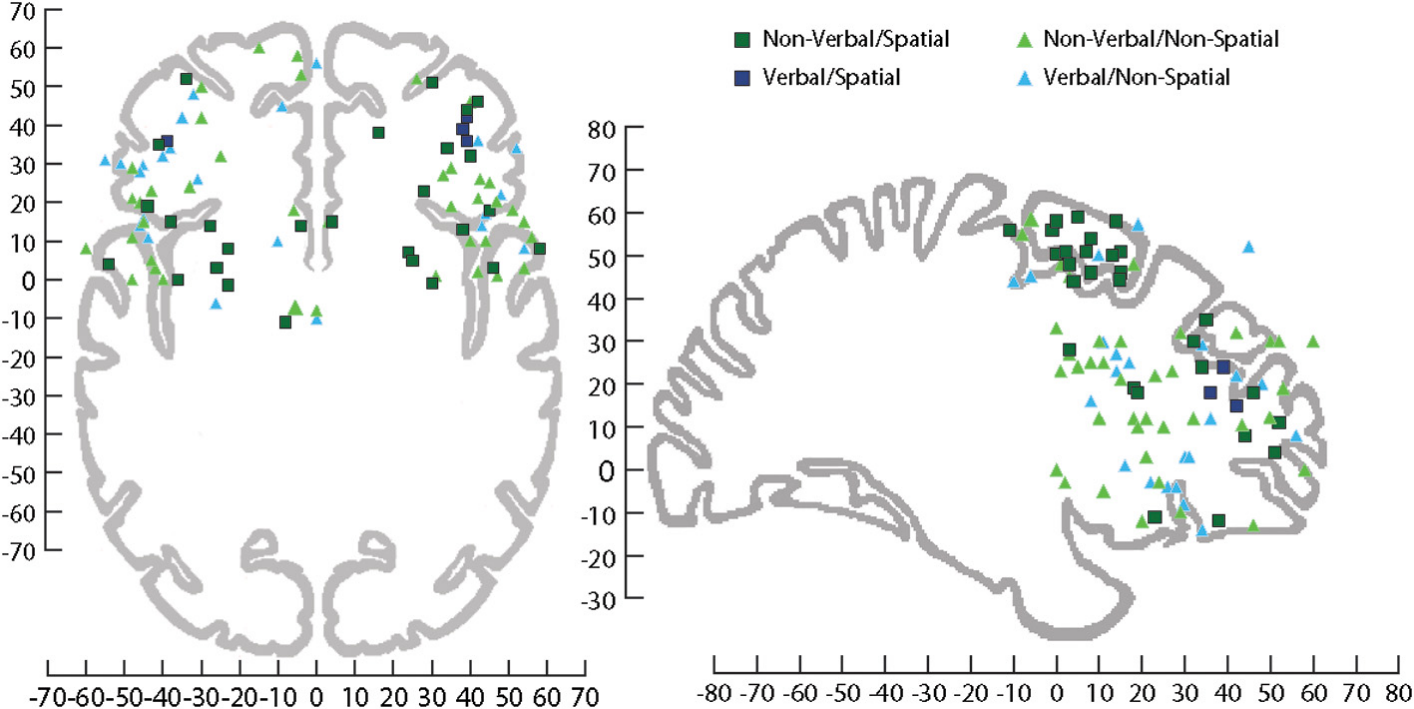
**Sagittal and horizontal view of human brain on which activations have been plotted across several fMRI studies involving processing of information during tasks that utilized stimuli that were Non-verbal/Spatial (dark green square), Non-verbal/Non-spatial (light green triangle), Verbal/Spatial (dark blue square), and Verbal/Non-spatial (light blue triangle)**. fMRI coordinates are plotted in Talairach space. Sagittal (*x* = +29 mm) and horizontal (*z* = −4 mm), Talairach images for reference from Talairach and Tournoux (1988). For a list of the studies plotted see (Supplemental Table 1).

Localization of auditory cognition to discrete networks in the human brain is complicated by the potential activation of language networks when verbal stimuli are used as memoranda in cognitive tasks. Comparing studies when verbal and nonverbal stimuli have been used has revealed activation in both DLPFC and VLPFC including the middle frontal gyrus and the anterior and posterior portions of the IFG. As might be predicted, VLPFC is active for language related functions but VLPFC activation also occurs for simple nonverbal auditory target detection/discrimination with tones (Linden et al., 1999; Kiehl et al., 2001; Muller and Basho, 2004; Huang et al., 2012), animal cries (Maeder et al., 2001), and melodies (Zatorre et al., 1994). Conversely, verbal discrimination has activated DLPFC (middle frontal gyrus) (Pedersen et al., 2000). This suggests that the prefrontal cortex is not simply dividing the processing of auditory information based solely on verbal information (Figure 6).

In order to examine auditory function independent of language circuits, noise bursts were used for both a spatial (localization) and non-spatial (pitch discrimination) auditory task (Alain et al., 2001). As predicted by the dorsal/ventral streams model, pitch processing evoked greater activation in the IFG while localization evoked greater activity in the superior frontal gyrus (Alain et al., 2001). The use of identical auditory stimuli under different demands, which led to diverse activation patterns, indicates cognitive load can recruit specialized areas within the frontal cortex (Alain et al., 2001). A similar pattern of results emerged in Du et al. (2013). In this study, subjects were trained to discriminate simultaneously presented vowel sounds. Vowels were presented with different frequencies or from different locations; this information was irrelevant for correct performance, but served as implicit information. After training, participants were exposed to both spatial and pitch differences while making vowel judgments and improved accuracy of vowel discrimination was observed when the pair of vowels presented matched their previous training (frequency or location). In addition, magnetoencephalography (MEG) activity was localized to the anterior ventral frontal regions for the group exposed to frequency changes, while MEG changes were more frequent in dorsal frontal regions for the group exposed to location changes (Du et al., 2013). Thus, even when participants did not make any explicit frequency or location choices the short term exposure to implicit spatial and object information segregated the dorsal and ventral prefrontal cortex respectively. This demonstrates that the activation of a particular neural network can be biased based on subtle cognitive demands.

In general, a division of labor for spatial and non-spatial information may exist (Ahveninen et al., 2013), and in non-human primates that do not possess language functions, may be most prominent. However, it is the underlying cognitive contingencies of a task that may ultimately recruit specific regions of frontal cortex. For example, pitch discrimination/ detection and auditory attention have been found to activate both DLPFC (Griffiths et al., 1998; Linden et al., 1999; Muller et al., 2001; Gaab et al., 2003; Seydell-Greenwald et al., 2013) and VLPFC Linden et al., 1999; Alain et al., 2001; Gaab et al., 2003; Seydell-Greenwald et al., 2013. Moreover, attention may bias which auditory network is recruited. Lipschutz et al. (2002) demonstrated that during dichotic listening when attention was divided both the lateral middle frontal gyrus and the IFG were activated although, the middle frontal gyrus was active when participants were told to selectively attend. Therefore, examining only whether a task has a spatial component is insufficient to determine which prefrontal regions will be recruited. Performing diverse types of cognitive processes such as making a pitch discrimination or dividing auditory attention may rely on different or overlapping auditory networks.

It has been questioned whether frontal lobe activity is related to cognitive demands or the stimulus properties within the task. Surprisingly, when verbal working memory is required, more dorsal regions (area 46/9) are recruited (Petrides et al., 1993; Petrides, 1996; Crottaz-Herbette et al., 2004). Whereas ventral regions (BA 47/12; 45) are utilized during active retrieval (Petrides, 1996; Kostopoulos and Petrides, 2008). In addition, areas of activation in frontal cortex can be shared by different auditory working memory demands (Arnott et al., 2005). It has also been suggested that within the auditory domain, DLPFC is more important for heavier memory loads, while VLPFC is necessary for dealing with attentional interference (Huang et al., 2013). Postle (2006) has reviewed the role of the prefrontal cortex with respect to information encoding, segregation, and manipulation of information, for visual working memory. Similar treatment needs to be given to the processing of auditory information and how dorsal and ventral prefrontal areas contribute to its encoding, manipulation, and short-term storage.

## Summary

The prefrontal cortex is involved in auditory cognition and receives information from a wide array of auditory regions including multisensory (STS) and unimodal auditory cortical regions. Understanding how that information is processed by the PFC and utilized during auditory cognition is an ongoing investigation. In the non-human primate, single-unit studies have indicated VLPFC has a specialized region for processing auditory stimuli but is also multisensory (Sugihara et al., 2006; Romanski, 2012) and involved in some aspects of higher auditory function (Cohen et al., 2007; Bizley and Cohen, 2013). In contrast, the DLPFC may have auditory responsive units but activity has mainly been observed during tasks requiring cognitive processes or localization of sound (Bodner et al., 1996; Kikuchi-Yorioka and Sawaguchi, 2000; Artchakov et al., 2007; Plakke et al., 2013a). Research in non-human primates suggests a functional division between DLPFC and VLPFC, with DLPFC utilized for spatial and auditory task requirements, while VLPFC is recruited for non-spatial and auditory feature processing as well as some cognitive operations. In humans, more cortical regions and cognitive ability complicate the picture. The spatial and non-spatial divide is somewhat supported; but new research suggests a more nuanced view is necessary and that different neural areas are recruited under various stimulus and cognitive demands. These recent neuroimaging studies provide support for a role of the prefrontal cortex in complex auditory cognition and demonstrate that attentional demands can shift which prefrontal network is activated. Overall, research from both humans and non-human primates suggests that the frontal cortex is essential in auditory cognition. Determining which specific cortical networks and prefrontal regions are critical in various aspects of auditory cognition is necessary for comprehending and treating communication disorders.

### Conflict of interest statement

The authors declare that the research was conducted in the absence of any commercial or financial relationships that could be construed as a potential conflict of interest.
