# Supplementary material for: Auditory connections and functions of prefrontal cortex
Source: Front Neurosci. 2014 Jul 23;8:199. doi: 10.3389/fnins.2014.00199 (PMC4107948; doi:10.3389/fnins.2014.00199)
Supplement: Supplementary file 1 [file DataSheet1.PDF]

Study	*N*	Imaging	Task Descriptions	Contrast	Verbal/ Non-Verbal	Spatial/ Non-spatial
Alain et al., 2001	15	fMRI	delayed match-to-sample based on angle	delayed match-to-sample based on pitch	NV	SP
Arnott et al., 2005	15	fMRI	noise bursts same/diff location	control: no memory requirement	NV	SP
Griffiths et al., 1998	3 and 6	fMRI and PET	listening to sound with percieved movement	listening to percieved stationary sound	NV	SP
Maeder et al., 2001	18	fMRI	response to concurrent audio coming from different locations	silence	NV	SP
Martinkauppi et al., 2000	10	fMRI	audiospatial 3-back	audiospatial 1-back	NV	SP
Weeks et al., 2000	9	PET	delayed matching auditory localization	silence	NV	SP
Zattore et al., 2002	10	2002	target sound localization	silence	NV	SP
Alain et al., 2001	15	fMRI	delayed match-to-sample based on pitch	delayed match-to-sample based angle	NV	NS
Arnott et al., 2005	15	fMRI	noise bursts pattern recognition	control: no memory requirement	NV	NS
Kiehl et al., 2001	10	fMRI	response to target stimuli	non-target stimuli	NV	NS
Linden et al., 1999	5	fMRI	response to target sine tone	non-target sine tone	NV	NS
Maeder et al., 2001	18	fMRI	response to animal cries	unusual sounds not animal cries	NV	NS
Muller et al., 2001	7	MRI	discrimintal tonal frequency glide countour	stable tone	NV	NS
			response to rising pitch	stable tone or opposite pitch glide	NV	NS
Platel et al., 1997	6	PET	familiar rhythm/melody	unfamiliar rhythm/melody	NV	NS
			discriminating pitch differences	stable pitch frequencies	NV	NS
Seydell-Greenwald et al., 2013	14	fMRI	counting the number of rising tones set in rising/falling tones	passive listening	NV	NS
Stevens et al., 2000	10	fMRI	silent count of target stimulus		NV	NS
Zatorre et al., 1998	20	PET	discriminating pitch intervals	passive listening to noise bursts	NV	NS
Zatorre et al., 1994	12	PET	passive listening to melodies	passive listening to noise bursts	NV	NS
			pitch comparison over time in a melody	passive listening to melodies	NV	NS

Lipschutz et al., 2002	10	PET	syllable discrimination by ear	identical syllables to both ears	VB	SP
			divided attention	Control: Repeat syllables	VB	SP
			selective attention	Control: Repeat syllables	VB	SP
Benedict et al., 1998	7	PET	auditory detection of syllables	Silence	VB	NS
Bennedict et al., 2002	12	PET	comined condition: attend to target syllables with button press and covert Attention mentally note target	Motor Only (button press) and Listen Only	VB	NS
Buchannan et al., 2000	10	fMRI	detect target emotional tone	detect target word	VB	NS
Burton et al., 2003	24	fMRI	target semantic or rhyming identification	target rhyming or semantic identification	VB	NS
Caplan et al., 2000	11	PET	semantic judgment of center embedded sentences	semantic judgement of right branching sentences	VB	NS
Du et al., 2013	24	MEG	identifying concurrent vowel sounds at different fundametnal frequencies	identifying concurrent vowel sounds at different angles	VB	NS
Griffiths et al., 1998	3 and 6	fMRI and PET	listening to sound with percieved movement	listening to percieved stationary sound	NV	SP
Hill and Miller 2010	16	fMRI	attending to particular location or pitch among multiple locations or pitches	no stimulus	VB	NS
Huang et al., 2013	18	MRI	target discrimination after cue in a list of letters	non-target letters	VB	NS
Kiehl et al., 2001	10	fMRI	response to target stimuli	non-target stimuli	NV	NS
Linden et al., 1999	5	fMRI	response to target sine tone	non-target sine tone	NV	NS
Lipschutz et al., 2002	10	ECAT HR+ Tomograph	syllable discrimination by ear	identical syllables to both ears	VB	SP
Maddock et al., 2003	8	fMRI	emotional word associations	no stimuli	VB	NS
Maeder et al., 2001	18	fMRI	response to concurrent audio coming from different locations	silence	NV	SP
			response to animal cries	unusual sounds not animal cries	NV	NS

Martinkauppi et al., 2000	10	fMRI	audiospatial 3-back	audiospatial 1-back	NV	SP
Muller and Basho 2004	7	fMRI	noun/verb semantic matching discrimintal tonal frequency glide countour	upper case/lower case visual matching	VB and NV	NS
			noun/verb semantic matching	upper case/lower case visual matching	VB	NS
Muller et al., 2003	9	fMRI	noun/verb semantic matching	upper case/lower case visual matching	VB	NS
Muller et al., 2001	7	MRI	discriminate tonal frequency glide countour	stable tone	NV	NS
Muller et al., 2001	7	MRI	response to rising pitch	stable tone or opposite pitch glide	NV	NS
Opitz et al., 2000	20	fMRI	verbally describing novel auditory stimulus	listening to pure tones	VB	NS
Pedersen et al., 2000	5	PET	word discrimination	silence	VB	NS
Petrides et al., 1993	10	PET	self ordered numbering	counting	VB	NS
			identify numbers left out of externally ordered set	counting	VB	NS
Platel et al., 1997	6	PET	familiar rhythm/melody	unfamiliar rhythm/melody	NV	NS
			discriminating pitch differences	stable pitch frequencies	NV	NS
Seydell-Greenwald et al., 2013	14	fMRI	counting the number of rising tones set in rising/falling tones	passive listening	NV	NS
Stevens et al., 2000	10	fMRI	silent count of target stimulus		NV	NS
Tranel et al., 2003	10	fMRI	naming common animals from their chacteristic sounds	sequential pitch countour judgement	VB	NS
Waters et al., 2003	18	PET/ERP	judgement of sentences containing subject object relative clauses	judgement of sentences containing object subject relative clauses	VB	NS

N =number of participants, NV=non-verbal, VB= verbal, SP=spatial, NS=non-spatial
